# PGSE, OGSE, and sensitivity to axon diameter in diffusion MRI: Insight from a simulation study

**DOI:** 10.1002/mrm.25631

**Published:** 2015-03-25

**Authors:** Ivana Drobnjak, Hui Zhang, Andrada Ianuş, Enrico Kaden, Daniel C Alexander

**Affiliations:** ^1^ Department of Computer Science and Centre for Medical Image ComputingUniversity College London (UCL)LondonUK

**Keywords:** diffusion imaging, diffusion gradients, axon diameter, oscillating gradient spin‐echo, pulsed gradient spin‐echo

## Abstract

**Purpose:**

To identify optimal pulsed gradient spin‐echo (PGSE) and oscillating gradient spin‐echo (OGSE) sequence settings for maximizing sensitivity to axon diameter in idealized and practical conditions.

**Methods:**

Simulations on a simple two‐compartment white matter model (with nonpermeable cylinders) are used to investigate a wide space of clinically plausible PGSE and OGSE sequence parameters with trapezoidal diffusion gradient waveforms. Signal sensitivity is measured as a derivative of the signal with respect to axon diameter. Models of parallel and dispersed fibers are investigated separately to represent idealized and practical conditions.

**Results:**

Simulations show that, for the simple case of gradients perfectly perpendicular to straight parallel fibers, PGSE always gives maximum sensitivity. However, in real‐world scenarios where fibers have unknown and dispersed orientation, low‐frequency OGSE provides higher sensitivity. Maximum sensitivity results show that on current clinical scanners (*G*_max_ = 60 mT/m, signal to noise ratio (SNR) = 20) axon diameters below 6 µm are indistinguishable from zero. Scanners with stronger gradient systems such as the Massachusetts General Hospital (MGH) Connectom scanner (*G*_max_ = 300 mT/m) can extend this sensitivity limit down to 2–3 µm, probing a much greater proportion of the underlying axon diameter distribution.

**Conclusion:**

Low‐frequency OGSE provides additional sensitivity to PGSE in practical situations. OGSE is particularly advantageous for systems with high performance gradients. Magn Reson Med 75:688–700, 2016. © 2015 The Authors Magnetic Resonance in Medicine published by Wiley Periodicals, Inc. on behalf of International Society for Magnetic Resonance in Medicine. This is an open access article under the terms of the Creative Commons Attribution License, which permits use, distribution and reproduction in any medium, provided the original work is properly cited.

## INTRODUCTION

Axon diameter statistics provide information about the function and performance of white matter pathways. Axon diameter directly relates to conduction velocity, the speed at which information propagates down neural pathways 1, 2. Hence imaging axon diameter could provide insight into basic brain operation as well as neuronal diseases that alter axon diameter distribution, such as autism 3, 4, amyotrophic lateral sclerosis 5, 6, or schizophrenia 7, 8. Developing a reliable technique to measure axon diameter in vivo is thus of great interest.

A number of techniques to measure axon diameter statistics using diffusion MRI have been proposed in the literature such as AxCaliber 9, 10, ActiveAx 11, 12, 13, double pulsed field gradient 14, 15, 16, and Q‐space imaging 17. These methods use either single pulsed field gradient, typically known as the pulsed gradient spin‐echo (PGSE) sequence, or use double pulsed field gradient which has been shown to have similar sensitivity to PGSE for pore size estimation at low diffusion weighting 18. However, various authors suggest that oscillating gradient spin‐echo (OGSE) offers benefits over PGSE for imaging pore sizes 19, 20, 21, 22, 23, 24.

A common argument is that high‐frequency OGSE sequences provide shorter effective diffusion time than PGSE and hence are able to probe smaller length scales. This is clearly an advantage for measuring the free diffusivity in porous systems with small pores because it minimizes the effects of restriction 25. However, it is not clear whether it is advantageous for measuring axon diameter where contrast at the long diffusion time limit may be more informative.

Recent results 20, 26 show that accurate microcapillary diameter estimates can be achieved with low‐frequency OGSE sequences or with a combination of high–low frequency profiles 24, suggesting that short diffusion times might not be necessary for estimating axon diameter. Furthermore, experiment design optimization algorithms 22, 27, which seek sequence parameters that maximize sensitivity to axon diameter, consistently produce a combination of high‐ and low‐frequency OGSEs together with the standard PGSE gradients. However, knowing the optimal solution alone does not provide a clear understanding of why the waveforms that emerge maximize the sensitivity.

More importantly, both simulation and phantom experiments so far have considered only idealized conditions, namely, the axons are perfectly parallel and the diffusion gradients are set to be perfectly perpendicular to the axons. In practice, however, axons typically are of unknown orientations; rather than strictly parallel to one another, their orientations are more often dispersed about one or more dominant orientations. Under such practical conditions, it is unclear whether sequences appropriate for the idealized conditions will remain adequate.

This article aims to provide a broad understanding of PGSE and OGSE signal sensitivity to axon diameter, to identify the most effective ways to maximize sensitivity in idealized and practical scanning situations. We use simulations of a standard two‐compartment white matter model (nonpermeable cylindrical axons) to investigate the signal sensitivity to axon diameter in detail. We consider parallel axons with known orientation as well as more realistic cases of unknown or dispersed orientation. Fiber dispersion refers to the fact that in practice axons in a voxel are never strictly straight and parallel to one another. Instead, there exists a continuous spread of orientations about one or more dominant orientation. This phenomenon is widespread in brain white matter, including the corpus callosum, one of the most coherently oriented structures 28, 29, 30.

The remainder of this article first specifies the signal models that we use in the article and defines the measure of sensitivity to axon diameter. Then we outline the simulation experiments and provide results that specify sensitivity factors for various scanning situations. We finish by summarizing the novel findings, limitations, practical predictions, and feasibility on current human imaging systems.

## METHODS

This section outlines the diffusion MR signal model for white matter, introduces the pulse sequences, and develops the concept of signal sensitivity to axon diameter we use here.

### MR Signal Model

We use two different models of white matter: the first one assumes parallel fibers and the second one assumes fiber dispersion. The first one is a simplified version of the CHARMED model 31, 32 described in 33. The model has two compartments, restricted and hindered, of populations of water molecules that each provides a separate MR signal. Restricted signal *S*_r_ comes from intra‐axonal water trapped inside parallel, nonabutting cylinders with equal diameter *a*, impermeable walls, and a fixed direction **n**. Hindered signal *S*_h_ comes from extra‐axonal water occupying the space outside the cylinders. The full model for the signal is

(1) $$S^{*}=S_{0}(fS_{r}+(1-f)S_{h})$$

where *S*_0_ is the MR signal with no diffusion weighting and f∈[0,1] is the proportion of water molecules inside the axons. The model for *S*_r_ is the Gaussian phase distribution (GPD) approximation 34 of the signal from particles trapped inside a cylinder, described and validated for OGSE sequences against Monte Carlo simulations in 35. The GPD approximation provides, in contrast to the short‐gradient‐pulse (SGP) approximation, good estimates of the signal for finite *δ*, which we focus on here. Intra‐axonal diffusion is unhindered along the axis of the cylinders. The model for *S*_h_ is the diffusion tensor model 36, i.e., anisotropic Gaussian distributed displacements, with diffusion coefficient $D_{\left|\right|}$ in the direction of the axons and $D_{⊥}$ in all perpendicular directions. The parallel diffusivity, $D_{\left|\right|}$, is the same as the intrinsic diffusivity inside the cylinders in the model for *S*_r_, following 33. A simple tortuosity model 37 sets $D_{⊥}=D_{\left|\right|}(1-f)$.

In the second model, we adopt the simplest possible model of fiber dispersion, the Watson distribution of orientations, as proposed in 12. The Watson distribution characterizes the fiber dispersion about the dominant orientation with a scalar parameter *κ*. For the most coherently oriented white matter, *κ* has a value about 32; for typical white matter, *κ* has a value about 8 or lower. All other parameters of this model, together with the 2‐compartment structure, are the same as in the model with parallel fibers described above.

### Pulse Sequences

**Figure 1:**
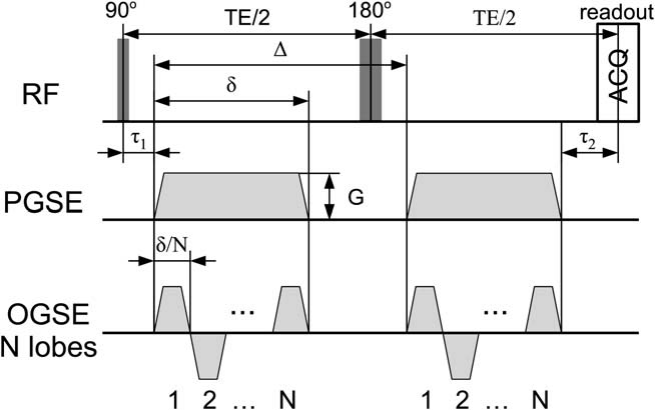
An illustration of a PGSE (top) and an OGSE (bottom) sequences showing all the variables. OGSE sequences are of trapezoidal shape with minimum achievable rise time to maximize diffusion weighting. The PGSE sequence is a special case of OGSE for *N* = 1.

Figure 1 illustrates the PGSE and OGSE sequences showing the set of variables for each. The PGSE sequence has the following variables: diffusion gradient pulse duration *δ*, time Δ from the beginning of the first (pre‐180) gradient waveform to the beginning of the second (post‐180), and gradient vector **G**. OGSE sequences have one additional variable: the number of lobes *N*. We include the time constant *τ*_1_ as the time between the middle of the RF pulse and the beginning of the first gradient waveform; *τ*_2_ as the time between the end of the second waveform and the readout at the centre of k‐space; *P*180 is the time required for the 180° RF pulse and accounts for the surrounding crusher gradients and additional time delays; and *P*90 is the duration of the 90° RF pulse.

Here we consider only trapezoidal OGSE sequences as we have shown previously 21, 38 that they maximize the sensitivity by maximizing the diffusion weighting for fixed time. We constrain *N* to be an integer number as is most typically used for OGSE methods 39, 40. When *N* = 1, the trapezoidal oscillating gradient reduces to PGSE sequence, hence we will hereafter refer to all sequences as OGSE. We calculate *b*‐value for these sequences as in 35 using:

(2) $$b=\frac{2|G{|}^{2}\gamma^{2}\delta^{3}}{15N^{2}}(5-\frac{15t_{r}N}{2\delta }-\frac{5t_{r}^{2}N^{2}}{4\delta^{2}}+\frac{4t_{r}^{3}N^{3}}{\delta^{3}})+|G{|}^{2}\gamma^{2}(\Delta -\delta ){\left(\frac{\left(1-{\left(-1\right)}^{N})(\delta -N\cdot t_{r}\right)}{2N}\right)}^{2}$$

where *t*_r_ is the rise time and *γ* is the gyromagnetic ratio.

### Sensitivity

We define the sensitivity of a measurement to axon diameter as a rate of signal change with axon diameter, i.e., the derivative $S^{*\prime }(a)$. Thus, the faster the signal changes with *a*, the more sensitive it is to axon diameter. From Eq. (1), we have:

(3) $$S^{*}\prime (a)=S_{0}fS_{r}^{\prime }(a).$$

Note that the hindered signal does not contribute to Eq. (3), as it does not depend on the axon diameter in the simple model (Eq. (1)). *S*_0_ in general depends on both pulse repetition time (TR) and echo time (TE). However, since *T*_1_ for white matter, typically around 500 ms, is much smaller than the typical TR values (about 10 s), hereafter, we simplify the equation for *S*_0_ by assuming infinite TR. We also normalize *S*_0_ by proton density, which gives $S_{0}=\exp(-\text{TE}(\delta ,\Delta )/T_{2})$, where $\text{TE}(\delta ,\Delta )=\delta +\Delta +\tau_{1}+\tau_{2}$ and *T*_2_ is the relaxation time of the white matter. Hence, here we calculate

(4) $$S^{*}\prime (a)=\exp(-\frac{\text{TE}(\delta ,\Delta )}{T_{2}})fS_{r}^{\prime }(a)$$

and use it as a measure of sensitivity of the full signal $S^{*}(a)$. Note that both $S^{*}(a)$ and $S^{*}\prime (a)$ are normalized by proton density. In this article, we compute $S_{r}^{\prime }$ using finite difference method with very fine diameter intervals to provide accurate approximation to the analytical derivative.

### Implementation

The simulations in this manuscript were performed using MISST sequence software toolkit 21, 35, 41 written in *Matlab*. They are very fast to compute and, on a standard desktop computer, it takes approximately 20 min to generate all the data used in the manuscript. The software is open source and can be downloaded from “https://www.nitrc.org/projects/misst.”

## RESULTS

This section contains simulation experiments that aim to identify key OGSE sequence parameters that maximize signal sensitivity to axon diameter, and evaluate the impact of these in both idealized and realistic conditions. Simulations investigate a wide space of sequence parameters Λ, feasible on current human imaging systems: G∈[0,300] mT/m ( G=|G|), δ∈[0,60] ms, Δ∈[δ+P180,100] ms, N∈[1,10]. We set typical values for time constants $\tau_{1}=10$ ms, $\tau_{2}=20$ ms, P180=10 ms, and slew rate SR = 200 T/m/s. Experiments use tissue models described in the Methods section and assume *f* = 0.7, $D_{\left|\right|}=1.7\times 10^{-9}$ m^2^/s 11, axon diameter a∈[0,10] µm and $T_{2}=70$ ms to match standard values in the white matter (at 3T). Note that *b*‐value, *q*‐value, and TE are not fixed and the sequence parameters are allowed to take any values in space Λ.

The first subsection maximizes the sensitivity of restricted signal $S_{r}^{\prime }(a)$ independently for all *a* defined above, over the wide space of sequence parameters Λ, and reduces Λ by identifying areas where sensitivity is negligible (close to zero). The second subsection includes *T*_2_ relaxation and investigates the sensitivity of the full signal as defined in Eq. (4). Both of these subsections assume that gradients and fibers are perfectly perpendicular, n⊥G, and no noise. The following subsections relax the perpendicularity condition and investigate sensitivity for a range of angles ∠(n,G) and fiber dispersion. The final subsection illustrates sensitivity for the different conditions in the presence of noise.

### Maximizing $S_{r}^{\prime }(a)$ for n⊥G

This section investigates the impact of OGSE sequence parameters on the restricted signal. We investigate $S_{r}^{\prime }(a)$ separately from *T*_2_ relaxation as it allows us to understand the fundamental dependencies of the tissue model on the pulse sequence parameters. An additional aim of this section is to reduce Λ by excluding the combinations where sensitivity is negligible. Here, we consider only the idealized case of n⊥G. As OGSE sequences are defined by four parameters, *G*, *δ*, Δ, and *N*, we aim to find in a systematic way which combination yields the largest $S_{r}^{\prime }(a)$.

### Choice of Δ

**Figure 2:**
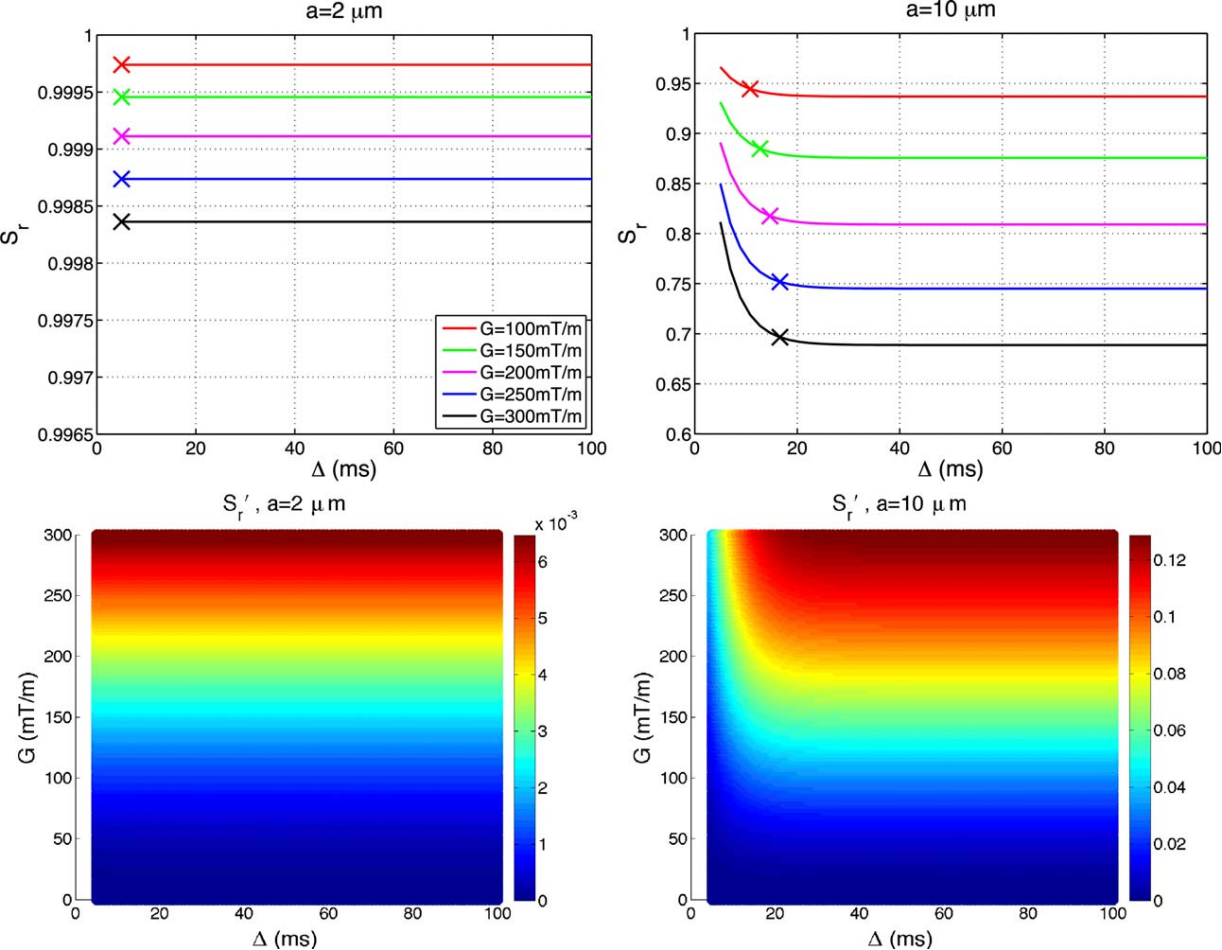
Impact of Δ on sensitivity of the normalized signal. Figure shows restricted signal $S_{r}(a)$ (top row) and its derivative $S_{r}^{\prime }(a)$ (bottom row) for a=2 µm (left column) and a=10 µm (right column) for a range of gradient strengths. Plots are generated with *δ* = 5 ms, and n⊥G. The absolute value of $S_{r}^{\prime }(a)$ for a particular combination of *G* and Δ is color coded, with dark red being the highest value. Crosses in the plots in the top row mark where signal flattens out to less than 1% difference with the changing Δ. Unit of $S_{r}^{\prime }(a)$ is 1/µm.

First, we assess the impact of different Δ's on $S_{r}^{\prime }(a)$. The experiment simulates restricted signal $S_{r}(a)$ and its derivative $\in_{r}^{\prime }(a)$ for the range of sequence parameters in Λ introduced above. Figure 2 shows $S_{r}(a)$ (top row) and $S_{r}^{\prime }(a)$ (bottom row) for a=2 µm (left column) and a=10 µm (right column). Data in the figure is shown for *N* = 1 (PGSE sequence) and *δ* = 5 ms (other values of *δ* produce similar effect). The absolute value of $S_{r}^{\prime }(a)$ for a particular combination of *G* and Δ is color coded, with dark red being the highest value, i.e., the largest $S_{r}^{\prime }(a)$.

Plots in the top row show restricted signals for a range of gradient strengths and share a common pattern. The signal initially decays for a few milliseconds, and then flattens out beyond a certain $\delta +\Delta_{0}$ which we mark with crosses in Figure 2. We define $\Delta_{0}$ as time such that beyond $\delta +\Delta_{0}$, the restricted signal change is below 1%. Plots show that the signal for a=2 µm (left) flattens out almost instantaneously, while for a=10 µm (right) it flattens after about 17 ms. These results are consistent with previous studies of signal from the restricted compartment as a function of diffusion time 42, 43. The flattening corresponds to the root mean squared displacement of free diffusion approaching the pore size, i.e., $\sqrt{2(\delta +\Delta_{0})D_{\left|\right|}}\approx a$. Bottom row of the figure shows that $S_{r}^{\prime }(a)$ initially increases and then also flattens after $\delta +\Delta_{0}$, suggesting that the influence of Δ on sensitivity is negligible beyond $\delta +\Delta_{0}$.

The value of $\Delta_{0}$ depends on OGSE parameters *G*, *δ*, and *N* for a given axon diameter (all other model parameters fixed). When maximized over the whole parameter space Λ, $\Delta_{0}=\{0,0,0,0,0,1.9,3.8,5.8,9.2,11.6\}$ms for axon diameters a∈{1,...,10} µm, respectively. For example, for a=1 µm, $\Delta_{0}=0$ showing that $S_{r}^{\prime }(a)$ flattens out immediately after the gradient pulse has finished for any *G*, *δ*, or *N* considered in this study. Conversely, for a=10 µm, $\Delta_{0}=11.6$ ms showing that $S_{r}^{\prime }(a)$ flattens out within at most 11.6 ms of the gradient pulse finishing.

The smallest Δ that maximizes sensitivity to axon diameter is thus in the range $\left[\delta +P180,\max(\delta +P180,\delta +\Delta_{0})\right]$. Typically P180≈10 ms, hence for the sequence parameters in Λ and axon diameter a∈[0,10] µm, $\Delta_{0}$ is almost always less than or comparable to *P*180. Hence, for the rest of this article, we set

(5) Δ=δ+P180

and refer to this Δ as optimal.

### Choice of *G*, δ, and N

**Figure 3:**
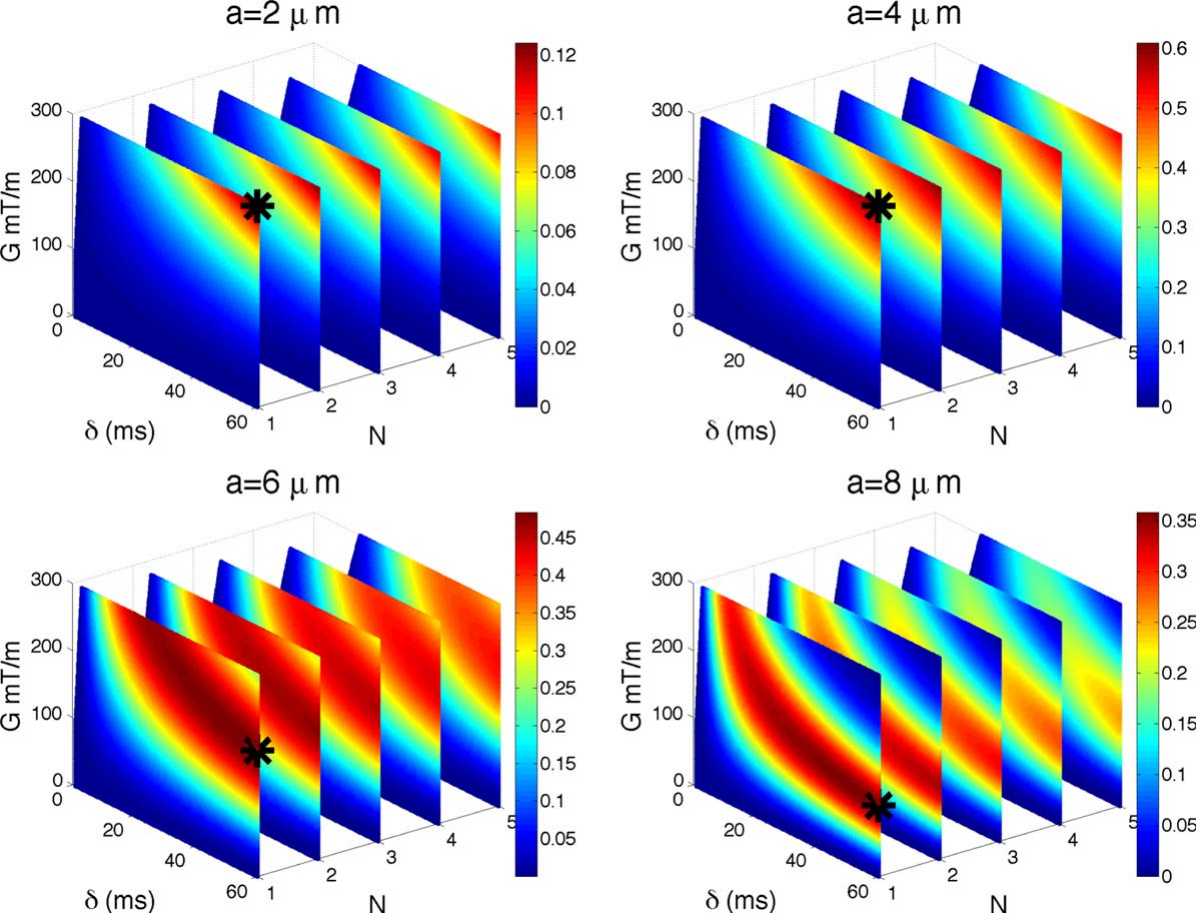
Impact of *G*, *δ*, and *N* on sensitivity. Figure shows $S_{r}^{\prime }(a)$ for a∈{2,4,6,8} µm and n⊥G. The absolute value of $S_{r}^{\prime }$ is color coded, with dark red being the highest value. Maximum intensity points are marked with a black star. Note that the plots are not perfectly rectangular due to excluded values of *δ* that did not satisfy the slew rate constraint. Unit of $S_{r}^{\prime }(a)$ is 1/µm.

Here we evaluate the impact of different gradient strengths *G*, gradient durations *δ*, and the number of lobes *N* on sensitivity $S_{r}^{\prime }(a)$. The experiment simulates $S_{r}^{\prime }(a)$ for the range of sequence parameters in Λ, but with Δ constrained according to Eq. (5). Figure 3 shows $S_{r}^{\prime }(a)$ for a∈{2,4,6,8} µm. We show absolute values of S′(a) for a particular combination of *G*, *δ*, and *N* and color code it, with dark red being the highest value. Absolute values are plotted because we are interested only in the magnitude and not in the sign of signal change.

Maximum intensity points are marked with a black star: (G,δ,N)= (300,60,1); (300,60,1); (187,60,1); (106,60,1) for a=2;4;6;8 µm, respectively. *G* is in mT/m and *δ* in ms. Values of *δ* which did not satisfy the slew rate constraint ( δ>G/2SR×N, SR is the slew rate) were excluded, hence the color‐coded plots are not perfectly rectangular. The figure shows that for a given axon diameter the maximum $S_{r}^{\prime }(a)$ is achieved for the minimum *N* = 1, which is the PGSE sequence (the first plane from the left). For a fixed *N*, maximum $S_{r}^{\prime }(a)$ is always achieved at maximum *δ*, but *G* may be less than maximum for larger *a*. These results describe the sensitivity of the normalized diffusion‐weighted signal, which is typically used in diffusion imaging experiments. The overall sensitivity varies with the axon size, however, they are all of the same order of magnitude.

This figure also shows that with low‐frequency oscillations, an order of magnitude lower *b*‐values than PGSE (*N* = 1) can achieve very similar sensitivity. For a fixed gradient strength *G* and pulse duration *δ*, *N* = 2 has very similar sensitivity to that of *N* = 1 (up to 0.01 µm^−1^ lower). As *N* increases, the sensitivity gets smaller, however, very gradually and *N* ={3,4} are still very close to *N* = 1, especially for a<7 µm. This shows that in the presence of strong restriction, a<7 µm, *b*‐value is not sufficient to explain signal attenuation and different combinations of *δ*, *G*, and *N* can produce the same *b*‐value while achieving very different signal attenuation and sensitivity to axon diameter; additional results in the Supporting Information illustrate this effect specifically.

### Maximizing $S^{*}\prime (a)$ for n⊥G

In this section, we investigate the effect of *T*_2_ relaxation on the previous findings. The main aim of this section is to maximize sensitivity $S^{*}\prime (a)$ of the full signal to axon diameter as in Eq. (4). We assume independence of *a* and *T*_2_.

Previous section shows that the optimal Δ in the absence of *T*_2_ relaxation is in the narrow range $\left[\delta +P180,\max(\delta +P180,\delta +\Delta_{0})\right]$. However, in the presence of *T*_2_ relaxation, increasing Δ increases TE, which—as given by Eq. (4)—will reduce the sensitivity of $S^{*}\prime (a)$. This favors short Δ even more strongly, so we retain the optimal setting for Δ as defined in Eq. (5).

**Figure 4:**
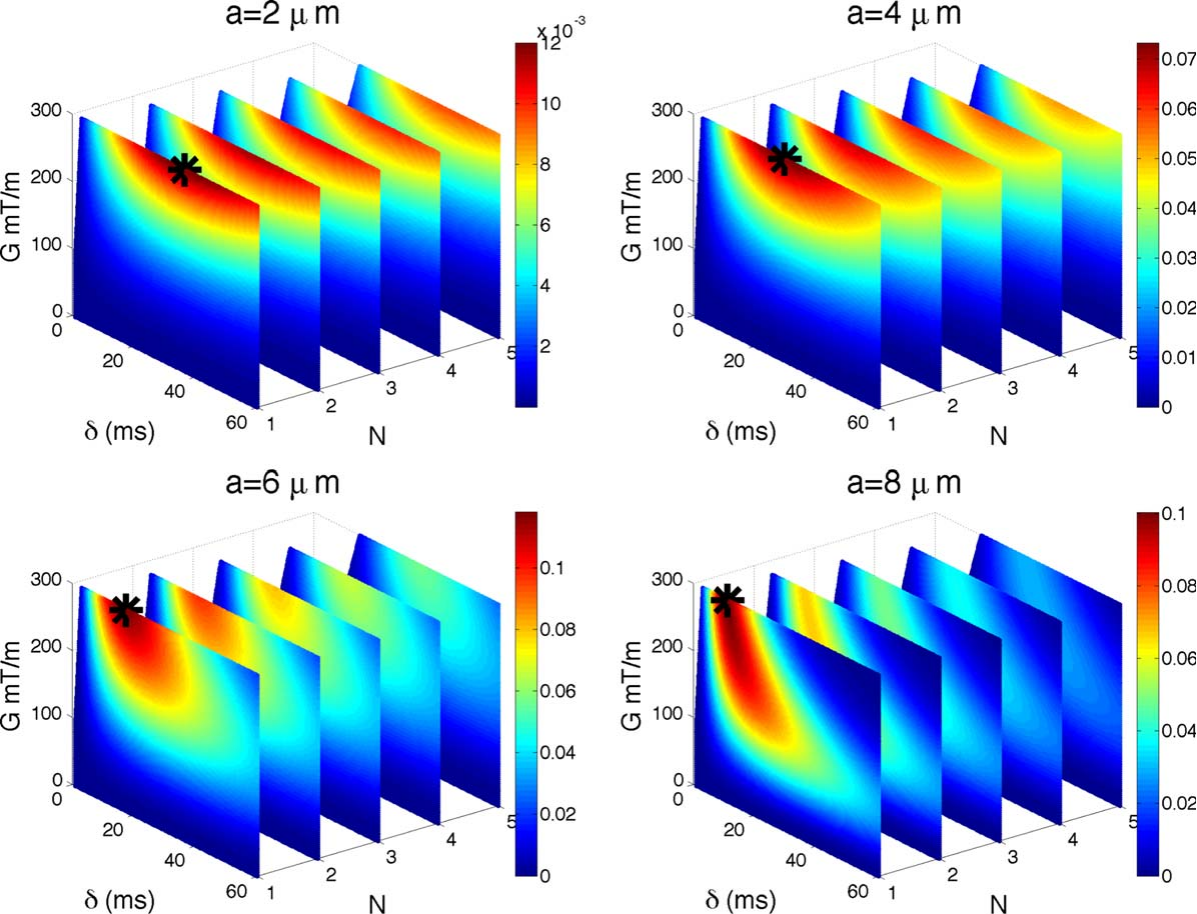
Impact of *T*_2_ decay on sensitivity. As Figure 3 but with $T_{2}=70$ms. Unit of $S^{*}\prime (a)$ is 1/µm.

The experiment simulates $S^{*}\prime (a)$ for the range of sequence parameters in Λ with Eq. (4). Figure 4 shows $S^{*}\prime (a)$ for a∈{2,4,6,8} µm. Maximum intensity points are marked with a black star: (G,δ,N)= (300,36,1); (300,29,1); (300,17,1); (300,11,1) for a=2; 4; 6; 8 μm, respectively. *G* is in mT/m and *δ* in ms.

The figure reveals two effects of the *T*_2_ decay on the sensitivity of the signal. First, it reduces *δ* compared with the case without *T*_2_ relaxation in the previous section. Second, the gradient strength increases to its maximum value to retain diffusion weighting, at least within the range of parameters feasible on human scanners Λ that we are interested in here, and for the axon diameter range a∈[0,10] µm. The optimal choice of *N* = 1 is unaffected by the *T*_2_ relaxation.

For smaller diameters, the resulting *δ* is larger than the corresponding value for larger diameters, emphasizing the need for stronger diffusion weighting when probing smaller length scales. However, even with the optimal combination of pulse sequence parameters with current clinical scanner hardware, the overall sensitivity for smaller diameters ( a<3 µm) is an order of magnitude smaller than the peak sensitivity at 6 µm (can be seen from the maximum values on the color bars).

Although the finding that TE should be kept short and *δ* reduced comes as no surprise, the less obvious finding is that as *N* increases, the optimal *δ* increases as well, and this can be seen from Figure 4 and Table 1 (in Section Practical Sensitivity in the Presence of Noise). By comparing the performance of optimal solutions between the sequences, we find which sequence would be preferred in different conditions. Although there are no analytic formulae that can predict these optimal solutions, they can be reliably identified with numerical optimization 21, 33. From now on we assume that *T*_2_ relaxation is present in all experiments.

**Table 1:** The table shows optimal *δ* values (in ms) used to simulate signals in Figure 7.

	***G***	*N*				
	**(mT/m)**	**1**	**2**	**3**	**4**	**5**
n⊥G	60	35	36	37	38	39
	80	36	36	37	38	39
	150	36	37	38	40	41
	300	36	38	40	42	45
n⊥G, θ=10∘	60	21	30	33	35	37
	80	19	28	31	35	36
	150	13	23	26	31	33
	300	10	18	21	27	29
Fibre dispersion *κ* = 16	60	23	31	33	36	37
	80	21	29	32	35	37
	150	16	24	27	32	34
	300	13	20	24	29	32

They maximize sensitivity to axon diameter a∈[0,3] for various settings of *G*_max_, *a* and *N* for 1 n⊥G, 2 n⊥G,θ=10∘, and 3 dispersion model with *κ* = 16.

### Maximizing $S^{*}\prime (a)$ for n⊥G

This section investigates signal sensitivity when diffusion gradients are not perfectly perpendicular to the fibers: $∠(n,G)=90^{o}\pm \theta$, where *θ* measures the deviation from the orthogonality. The *T*_2_ relaxation is included and the experiment simulates sensitivity $S^{*}\prime (a)$ of the full signal for a range of $\theta \in [0^{\circ },45^{\circ }]$, sequence parameters in Λ, and Δ as in Eq. (5).

**Figure 5:**
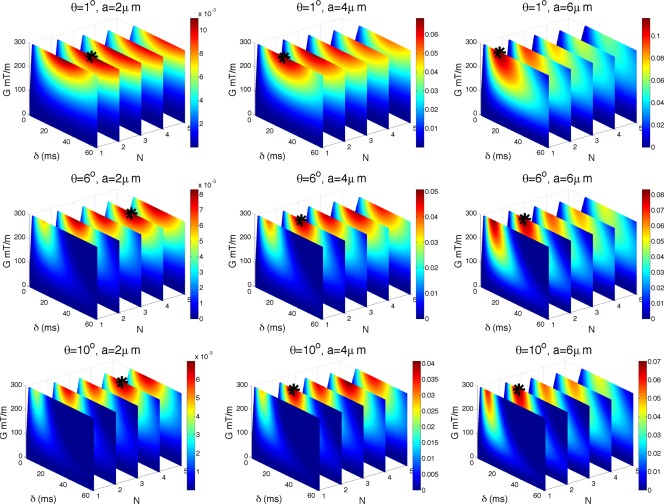
Impact of *θ* on sensitivity. As Figure 4 but for $\theta =1^{o}$ (top), $\theta =6^{o}$ (middle), $\theta =10^{o}$ (bottom). Unit of $S^{*}\prime (a)$ is 1/μ m.

Figure 5 shows $S^{*}\prime (a)$ for a∈{2,4,6} µm and $\theta \in \{1^{\circ },6^{\circ },10^{\circ }\}$. The choices for *θ* are motivated by the practical constraints on the number of gradient directions used in HARDI acquisitions. As the number of gradient directions is finite, some fibers may not have a single gradient direction that is close to perpendicular to it. For typical HARDI experiments with 30, 60, or 90 directions, the worst cases correspond to $\theta =10^{\circ },6^{\circ },4^{\circ }$, respectively. Maximum intensity points are marked with a black star: (G,δ,N)= [(300,36,2) (300,26,1) (300,17,1)]; [(300,32,4) (300,22,2) (300,20,2)]; [(300,27,4) (300,18,2) (300,17,2)] for (θ,a)=[(1, 2) (1, 4) (1, 6)]; [(6, 2) (6, 4) (6, 6)]; [(10, 2) (10, 4) (10, 6)], respectively. *G* is in mT/m, *δ* in ms, *θ* in degrees, and *a* in μm. The optimal combination of *G* and *δ* stays relatively similar to the perpendicular case with *G* maximized and *δ* reduced to keep TE short. However, the choice of *N* is different.

The figure shows that *N* = 1 cases are very much affected by the nonperpendicularity, and sensitivity drastically drops as *θ* increases. For example, for a=2 µm, we have that $S^{*}\prime (2)=0.012$ ( n⊥G) drops to 0.010 ( $\theta =1^{\circ }$), 0.005 ( $\theta =6^{\circ }$), and 0.003 ( $\theta =10^{\circ }$). Conversely, *N* > 1 cases are much less affected and preserve their sensitivity better. In the same example as above, *N* = 2 has $S^{*}\prime (2)=0.0113$ ( n⊥G) then drops to 0.011 ( $\theta =1^{\circ }$), 0.007 ( $\theta =6^{\circ }$), and 0.006 ( $\theta =10^{\circ }$), which indicates a rate of sensitivity loss approximately two times slower than *N* = 1 case. Hence, as *θ* increases *N* > 1 has higher sensitivity than *N* = 1.

The main reason for this is that as *θ* increases the proportion of the signal from free diffusion parallel to the fibers increases, and hence sequences with high *b*‐value (such as the *N* = 1 sequences from above) attenuate the signal much more significantly and reduce the sensitivity. The normalized diffusion signal from water trapped in a cylinder $S_{r}(a)=S_{r||}S_{r⊥}(a)$, is the product of components arising from displacements parallel $S_{r||}$ (free diffusion) and perpendicular $S_{r⊥}$ (with restriction) to the main axis as described in 32. The sensitivity, which is a simple derivative as defined in Methods section, is hence $S_{r}^{\prime }(a)=S_{r||}S_{r⊥}^{\prime }(a)$. Therefore, although the diffusion parallel to the main axis of the cylinder does not depend on its diameter *a*, it directly influences the sensitivity through this product.

As a result, *N* > 1 sequences, for a given *G* and *δ*, can have higher sensitivity than *N* = 1 sequence. *N* > 1 sequences have an order of magnitude lower *b*‐values than *N* = 1 sequence, and hence their parallel component $S_{r||}$ is much larger than that of *N* = 1 case. However, the perpendicular component depends much less strongly on the *b*‐value and $S_{r⊥}^{\prime }(a)$ is very similar for *N* > 1 and *N* = 1 cases as discussed and shown in Figure 3 in the n⊥G section. Consequently, as *θ*, and hence the influence of parallel signal component increases, sensitivity $S_{r}^{\prime }(a)$ of *N* > 1 becomes larger than that of *N* = 1 sequence (an illustrative example is shown in the Supporting Information). This effect is most influential for smaller axon diameters for which the optimal *δ* is longest leading to very high *b*‐values in *N* = 1 sequences. As the axon diameter increases, *δ* reduces. As a result, for sufficiently large axon diameters ( a>7 µm), *N* = 1 maintains its advantage over *N* > 1 seen for n⊥G.

### Maximizing $S^{*\prime }(a)$ for Fiber Dispersion

**Figure 6:**
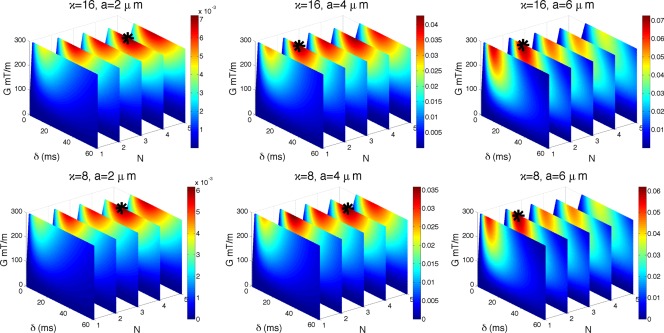
Impact of fibre dispersion on sensitivity. Figure shows $S^{*}\prime (a)$ for *κ* = 16 (top row) and *κ* = 8 (bottom row). a∈{2,4,6} µm and $T_{2}=70$ ms. As in previous figures: the absolute value of $S^{*}\prime (a)$ is color coded, with dark red being the highest value; maximum intensity points are marked with black stars; plots are not perfectly rectangular due to the slew rate constraint. Unit of $S^{*}\prime (a)$ is 1/µm.

The experiment simulates $S^{*}\prime (a)$ for the model with fiber dispersion described in Methods section, and the range of sequence parameters in Λ, with Δ set according to Eq. (5). The principle orientation of the fibers is perpendicular to the gradient vector. Axon diameter is in the range a∈[0,10] µm and κ∈[0,32]. Figure 6 shows the sensitivity of the full signal $S^{*}\prime (a)$ (with *T*_2_ relaxation) for a∈{2,4,6} µm and κ∈{8,16}. Maximum intensity points are marked with a black star: (G,δ,N)= [(300,29,4) (300,20,2) (300,18,2)]; [(300,26,4) (300,27,4) (300,17,2)] for (κ,a)=[(16, 2) (16,4) (16, 6)]; [(8, 2) (8, 4) (8, 6)], respectively. *G* is in mT/m, *δ* in ms, and *a* in μm.

**Figure 7:**
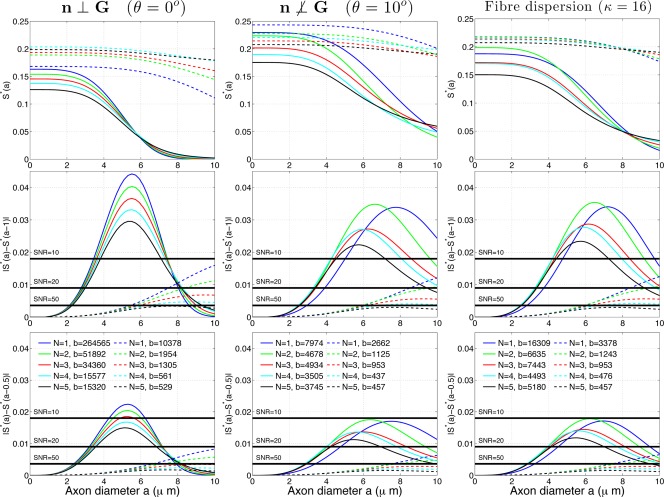
Simulation results for *G* = 300 mT/m (full lines), and *G* = 60 mT/m (dashed lines) for n⊥G (left column), n⊥G,θ=10∘ (middle column), and dispersion model with *κ* = 16 (right column). The top row shows full diffusion signal $S^{*}(a)$ (normalized with proton density) the middle row shows 1 µm resolution in axon diameter, and the bottom row shows 0.5 µm resolution. Each curve corresponds to a sequence setting optimized for a particular condition (each column) and for a particular choice of *N* (different colors). *b*‐value is in ${s/\text{mm}}^{2}$.

The optimal combination of *G* and *δ* stays relatively similar to the perpendicular case (as in the previous section) with *G* maximized and *δ* reduced to keep TE short. However, similarly as in the previous section, the choice of *N* is different. For both values of *κ* and diameters below approximately 7 µm, *N* > 1 enhances sensitivity over *N* = 1. The effect of fiber dispersion is similar to that of nonperpendicular gradients described in the previous section. High *b*‐values in *N* = 1 sequences attenuate the signal very rapidly because of unrestricted displacements parallel to the gradient direction. The larger the dispersion, i.e., smaller *κ*, the larger the benefits of the *N* > 1 sequences. Again, for the sufficiently large axon diameters ( a>7 µm), *N* = 1 maintains its advantage seen in the case of n⊥G.

### Practical Sensitivity in the Presence of Noise

This section evaluates whether the sensitivity enhancement of *N* > 1 over *N* = 1 shown in the previous two sections, is significant enough to make a practical difference in the presence of noise. To illustrate practically achievable sensitivities, we compare the signal difference due to a change *ϵ* in axon diameter,

(6) $$\left|S^{*}(a)-S^{*}(a-\epsilon )\right|,$$

to the standard deviation of the noise *σ*. *ϵ* is determined by the axon diameter resolution we are interested in. For example, ϵ=1 µm would allow us to assess whether we can distinguish 1 and 2 μm or 2 and 3 μm etc. It is worth noting that we can not use the slope $S^{*}\prime (a)$ as we did previously, because $S^{*}(a)$ is nonlinear and the slope can vary considerably over any finite interval *ϵ*. Equation (6) corresponds to the integration of $S^{*}\prime (a)$ over the interval [a−ϵ,a].

We compare the best *N* = 1 signal, against the best *N* > 1 signals for maximizing sensitivity to the smallest diameters in the previously introduced cases (1) n⊥G, (2) n⊥G,θ=10∘, and (3) dispersion model with *κ* = 16 for comparison. We choose $\theta =10^{\circ }$ as it corresponds to the worst case for HARDI with 30 directions. We choose *κ* = 16, which corresponds to typical values in brain's corpus callosum 12.

Parameters for the most sensitive sequences for diameters a≤3 µm are extracted from results in previous sections presented in Figures 4, 5, 6 and are summarized in Table 1. The optimal gradient strength for maximizing sensitivity is always the maximum possible $G=G_{\max}$ and the diffusion time Δ is set as in Eq. (5). Each *G*, *N*, and *a* combination has a corresponding *δ*. We average *δ*'s for a∈{1,2,3} µm (variation within 2 ms for different *a*'s) to obtain a single *δ* for each *G* and *N* combination.

The experiment simulates the full signal $S^{*}(a)$ (with *T*_2_ relaxation) for three different settings of standard deviation of noise *σ*: σ∈{0.018,0.009,0.0036} corresponding to the SNR ∈{10,20,50} of unweighted diffusion signal, respectively, at base setting of TE_0_ = 120 ms. These were calculated using $\sigma =\exp(-{\text{TE}}_{0}/T_{2})/\text{SNR}$. We use realistic model parameters outlined at the start of the Results section, *f* = 0.7, $D_{\left|\right|}=1.7\times 10^{-9}m^{2}/s,D_{⊥}=0.51\times 10^{-9}m^{2}/s$ (from the tortuosity model), axon diameter a∈[0,10] µm and the range of *G* and *N* parameters in Λ, with Δ fixed as in Eq. (5).

Figure 7 shows simulation results for *G* = 300 mT/m (full line) and resolution ϵ∈{0.5,1} µm. The straight black lines marked with SNR are the *σ* values calculated previously (see paragraph above). The top row shows $S^{*}(a)$, middle row shows $\left|S^{*}(a)-S^{*}(a-1)\right|$, and the bottom row shows $\left|S^{*}(a)-S^{*}(a-0.5)\right|$ for all a∈[0,10] µm. The top row illustrates visually the different signals. Some signals change faster with the changing *a* (i.e., have faster decay) and hence are more sensitive to axon diameter. For example in the top left, the *N* = 1 signal (in blue) decays the fastest, i.e., is the most sensitive to axon diameter. Conversely, *N* = 5 (in black) is the slowest, and hence is the least sensitive one. Top middle and right plot show that the signals are decaying slower when n⊥G or when there is fiber dispersion. In line with the previous sections, for a∈[0,6] µm, their ordering regarding the sensitivity has also changed, with *N* > 1 signals having faster decay than the *N* = 1 signal. The figure also shows that large *b*‐value does not guarantee large sensitivity, and can reduce sensitivity when n⊥G or when there is fiber dispersion. For example, in the plots with dispersion, the blue plot which has the largest *b*‐value, has least sensitivity. The middle row of Figure 7 illustrates feasibility of 1 μm resolution for different noise levels. For example, the *N* = 1 signal in the first column has 1 µm resolution in ranges a∈[3.4,7.5] µm when SNR=10, a∈[2.8,8] when SNR = 20 and a∈[2.2,8.2] when SNR = 50. *N* > 1 sequences do a bit worse, with *N* = 2 being very close to *N* = 1. Middle and right columns for a∈[0,6] show a different trend: sensitivity of *N* = 1 is less than that of *N* > 1, with *N* = 4 doing the best. The bottom row of Figure 7 shows results for ϵ=0.5 µm resolution in axon diameter. The main trends are similar to the ϵ=1 µm case, just the range of axon diameters for all *N* reduces. For example, the *N* = 1 signal in the first column has 0.5 µm resolution in ranges a∈[4.2,6.2] for SNR=10, a∈[3.2,7.1] µm for SNR=20 and a∈[2.5,8] for SNR = 50.

Figure 7 also shows results for *G* = 60 mT/m (dashed line). All sequences are much less sensitive to changes in axon diameter, especially to small diameters, as can be seen from the middle and bottom rows. For example, the *N* = 1 signal in the left column cannot detect 1 µm resolution or below when SNR ≤10 or 0.5 µm resolution or below when SNR ≤20, which is much worse than for the stronger gradient strength case. *N* > 1 signals do slightly better for the small diameters ( a<6 µm) when $\theta =10^{\circ }$ or fiber dispersion, however, the advantage is significantly less prominent than for the *G* = 300 mT/m case. This can be seen from comparing the green and the blue dashed line in the right column which are now much closer to one another.

In addition to the diameter resolution, another quantity of interest is the smallest axon diameter one can distinguish from zero, which we denote as *a*_0_. We can determine *a*_0_ by setting ϵ=a for any gradient strength, SNR, and *N*. Table 2 shows *a*_0_, for a range of gradient strengths G∈{60,80,150,300} mT/m and SNR ∈{10,20,50}. The top four rows of the table show the case when n⊥G, the middle four rows when n⊥ G,θ=10∘ and the bottom four when fibers are dispersed with *κ* = 16. The shaded values in the table are the lowest possible *a*_0_ across different N∈{1,2,...,5}, for each combination of *G* and SNR.

**Table 2:** The table shows $a_{0}(\mu$m) for a range of *G* and SNR (10, 20 and 50).

		***N* = 1**			***N* > 1**		
	G (mT/m)	10	20	50	10	20	50
n⊥G	60	7.2	6.0	4.6	7.5 (*N* = 2)	6.1 (*N* = 2)	4.7 (*N* = 2)
	80	6.2	5.1	4.0	6.4 (*N* = 2)	5.2 (*N* = 2)	4.1 (*N* = 2)
	150	4.5	3.7	3.0	4.5 (*N* = 2)	3.8 (*N* = 2)	3.0 (*N* = 2)
	300	3.2	2.7	2.1	3.2 (*N* = 2)	2.7 (*N* = 2)	2.1 (*N* = 2)
n⊥ G,θ=10∘	60	7.8	6.5	5.0	7.7 (*N* = 2)	6.3 (*N* = 2)	4.9 (*N* = 2)
	80	6.9	5.7	4.5	6.7 (*N* = 2)	5.4 (*N* = 2)	4.2 (*N* = 2)
	150	5.3	4.4	3.5	4.9 (*N* = 2)	4.1 (*N* = 2)	3.2 (*N* = 2)
	300	4.3	3.5	2.8	3.7 (*N* = 4)	3.1 (*N* = 4)	2.4 (*N* = 4)
Fibre dispersion *κ* = 16	60	7.7	6.4	5.0	7.7 (*N* = 2)	6.3 (*N* = 2)	4.9 (*N* = 2)
	80	6.8	5.7	4.4	6.7 (*N* = 2)	5.4 (*N* = 2)	4.2 (*N* = 2)
	150	5.2	4.4	3.4	4.9 (*N* = 4)	4.1 (*N* = 4)	3.2 (*N* = 4)
	300	4.1	3.4	2.7	3.7 (*N* = 4)	3.0 (*N* = 4)	2.4 (*N* = 4)

*a*_0_ represents the smallest axon diameter below which one cannot distinguish from zero. The shaded values are the lowest possible *a*_0_ across different *N*, for each combination of *G* and SNR.

The table shows that when n⊥G the lowest *a*_0_ is achieved for *N* = 1. Conversely, when n⊥ G or fibers are dispersed, the lowest *a*_0_ is achieved for *N* > 1, with up to 1–2 μm difference. Differences between the *N* = 1 and *N* > 1 increase with larger *G*. At 60 mT/m, the difference is negligible, while for 300 mT/m it goes to 1–2 μm. Hence, the impact of using *N* > 1 is larger for larger *G*.

**Figure 8:**
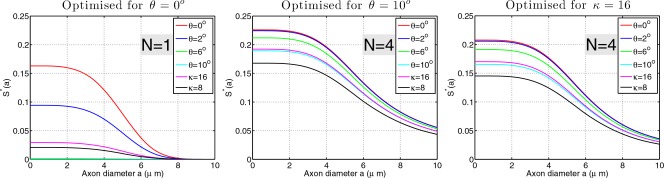
Stability of maximal sensitivity sequences across different scenarios. *G*_max_ = 300 mT/m. The set of signal curves in each plot are computed with the identical sequence setting determined by optimizing for one of the three scenarios: n⊥G,n⊥G(θ=10∘), fiber dispersion (*κ* = 16). The optimal choice of *N* for each scenario is shown.

Finally, we investigate how well the sequence optimized for one particular condition can cope with the other conditions. Figure 8 shows the results for the optimal sequences extracted from Figure 7 for three different scenarios: (1) n⊥G, (2) n⊥ G,θ=10∘, and (3) dispersion model with *κ* = 16, and how each of these performs for $\theta \in \{0^{\circ },2^{\circ },6^{\circ },10^{\circ }\}$ and κ∈{8,16}. Results are shown for *G*_max_ = 300 mT/m.

The figure shows that, although *N* = 1 sequence is maximally sensitive to small diameters in the idealized scenario (*θ* = 0), when θ>0 or in the presence of dispersion, the signal suffers greatly, leading to dramatic loss in the sensitivity. Conversely, *N* = 4 sequences are much less affected by the changes in *θ* and *κ* showing much more stability over different conditions. In the practical scenario, where one single sequence has to be chosen and applied to the whole brain, this stability across different *θ*'s and *κ*'s is very important.

## DISCUSSION

In this article, we explore, using simulations and a simple model of nonpermeable cylindrical axons, the optimal combinations of OGSE sequence settings for sensitivity to axon diameter in various situations. We find that when gradients are perfectly perpendicular to the fibers, maximum sensitivity is always achieved for *N* = 1 (a standard PGSE sequence), maximum possible *δ* and some *G* that depends on axon diameter *a*. However, in practice, we often do not know the fiber orientation; even if it is known, there is likely orientation dispersion about the dominant direction. Further simulations show that in either case, the maximum sensitivity is achieved for an *N* > 1, at least when a<7 µm, which represents the majority of axon diameters in the human corpus callosum 44. *N* > 1 is advantageous in these situations because the oscillating waveforms can achieve high sensitivity at a modest *b*‐value, which in turn enables OGSE sequences to retain their sensitivity for unknown fiber orientations and in the presence of dispersion by avoiding excessive signal attenuation from unrestricted displacements in the fiber direction. We show that this is particularly advantageous for systems with high performance gradients.

### The Role of Low‐Frequency OGSE

One of the novel findings is that the OGSE sequences that maximize sensitivity generally have low frequency. The traditional view has been that a high‐frequency OGSE increases sensitivity to pore sizes because it has a shorter effective diffusion time. Similarly for PGSE, we find that there is no need for very short diffusion times and, in the absence of *T*_2_ relaxation or when TE is fixed, the best choice of *δ* is as long as possible.

This finding does not contradict the earlier optimization results that produced high‐frequency oscillating gradients for maximizing the sensitivity to axon diameter 21, 22, 27, 38. The high‐frequency oscillations appear because the optimization maximizes sensitivity to the intrinsic diffusivity parameter in addition to the axon diameter. To estimate both parameters simultaneously requires two distinct measurements and the best choice for the second measurement is the high‐frequency OGSE. The smaller spatial scale is achieved with the high‐frequency pulses, we consistently obtained previously 21, 22, 27, 38. The frequency of these oscillations additionally depends on the angle between the gradient and the fibers, and this is something we will be looking at in the future.

The key advantage of OGSE in practical situations with unknown and/or dispersed fiber orientation is that it retains sensitivity to axon diameter from perpendicular displacements while reducing sensitivity to parallel displacements that can attenuate the signal fully from high *b*‐value PGSE measurements.

### Implications for Practical Applications

In practical applications, e.g., for a realistic tissue model with fiber dispersion or unknown orientation and realistic SNR, the choice of *N* impacts significantly the range of axon diameters that can be identified and distinguished from one another. For a fixed TE, sequences with *N* > 1 increase the detectable difference in the signal, and distinguish between axon diameters that *N* = 1 cannot. For example, for $\theta =10^{\circ }$, 300 mT/m scanner's lower limit for SNR = 10 is approximately 4.3 µm for *N* = 1, and 3.1 µm for *N* = 4. In another example, Figure 7 middle row shows that for $\theta =10^{\circ }$ and *G* = 300 mT/m, *N* = 4 signal can with 1 µm resolution access axon diameters which are 1 µm lower than with *N* = 1. Hence, going from *N* = 1 to the best choice of *N* increases the overlap of the range of axon diameter sensitivity with naturally occurring axon diameters, which can potentially allow more accurate axon diameter imaging of in vivo human brain. These advantages are especially prominent on stronger gradient strengths.

It is also worth noting that moving from the traditional PGSE sequences to the OGSE sequences discussed here is simple and offers additional practical benefits. They have only one additional parameter, the number of lobes *N*, therefore, are easy to implement and run on standard clinical and preclinical scanners. Low‐frequency OGSE (*N* > 1) can also significantly reduce the gradient heating and deal better with eddy currents than PGSE sequences.

### Model Considerations

In this work, we choose a prevailing simple model of white matter 11, 12. More complex models increase the difficulty of locating most sensitive sequence parameters and could potentially influence some of the results presented here, e.g., membranes with permeable walls could create an increase in signal loss for sequences with large *b*‐value, hence OGSE sequences with *N* > 1 would have further advantage over PGSE sequences. Another simplification of our model is the representation of axons as a collection of cylinders with one diameter. It is known from histology 44 that in the brain, white matter axons have a diameter distribution which is often modelled as a Gamma function. Nevertheless, an understanding of which sequence is most sensitive to each particular axon diameter is very informative as it provides a deeper understanding of the relationship between individual restrictions and sequence parameters, and is a first step toward understanding sensitivity to the combinations of different diameters.

For the extracellular space, we use a simple tortuosity model which has been previously used for estimating axonal indices in the human brain 11, 28. In some situations that model can become inaccurate, for example, if extracellular space exhibits restricted diffusion as observed experimentally by 45 or at long diffusion times where the diffusion constant may become time dependent 46, 47. A future challenge will be to include this in our modelling of the extracellular space. Assuming weak dependence of the extracellular space on the axon diameter, our results should not be significantly affected. However, significant extracellular space restriction would disrupt axon diameter estimation, as it is indistinguishable from axonal restriction, so our results could change, albeit in a way which is very hard to predict without actually doing the modelling. This nontrivial problem is the topic of ongoing research.

We assume independence of the axon diameter and the *T*_2_‐signal. A recent study 48 has suggested that smaller axons might have a shorter *T*_2_‐relaxation time. Increasing *T*_2_ value favors even more the OGSE sequences, while reducing it does the opposite. However, for these effects to be significant, *T*_2_ needs to change dramatically. Varying *T*_2_ within the 17 ms window reported in 48, or even double that, creates almost no difference in our results, e.g., up to 2 ms difference in the current optimal *δ*'s, and hence no difference in our conclusions.

Here we use the diffusion coefficient $D_{\left|\right|}=1.7\times 10^{-9}$ m^2^/s, which is often used for in vivo white matter simulations 11. When using a diffusion coefficient that is larger, smaller axons become even more difficult to detect. For example, if the diffusion coefficient is as high as in pure water ( $3\times 10^{-9}$ m^2^/s), the sensitivity range shifts toward higher axon diameters by approximately 1 µm. In this case, OGSE sequences are even more beneficial, especially in the case of dispersion where, due to high diffusion coefficient, the signal loss is large and having lower *b*‐values provided by OGSE sequences is a big advantage. When using a diffusion coefficient that is smaller than the one we use here, e.g., as in ex vivo samples, the trend is opposite: smaller diameters are less difficult to measure, and the OGSE sequences less beneficial although still preferred over the PGSE in the case of dispersion.

We also set *P*180 to the typical value of 10 ms. However, different scanners may have different *P*180s and there could be additional necessary sequence components between the gradients. Larger *P*180 increases TE, so the optimal gradient duration could potentially be shorter, favoring the PGSE sequence. Shorter *P*180 does the opposite. Typical variations would change TE for a few milliseconds and hence would have very little effect on the results (1 or 2 ms difference in *δ*).

Finally, we note that here we consider only the model‐based approach to estimating axon diameters. Other approaches use, for example, the diffraction pattern in the signal 49, 50, 51, 52 to infer pore size, however, they require short‐gradient‐pulses and thus high gradient strengths, and are very difficult to observe in the clinical setting. Within that setting, which is our focus, the pulse width is always finite and gradient strength does not exceed 300 mT/m, hence we calculate the signal using the Gaussian Phase Distribution approximation, which under this condition agrees closely with very accurate solvers including the matrix formalism and Monte Carlo simulations 35.

## CONCLUSION AND PERSPECTIVES

This simulation study provides the theoretical foundation for understanding the importance of OGSE sequences in the estimation of cylindrical pore sizes. First, in the idealized situation in which the gradient is perfectly perpendicular to the pore axis, OGSE offers no benefit over PGSE. However, the OGSE provides benefits for model‐based diffusion MRI in practical situations where fiber orientations are unknown and/or dispersed. We demonstrate that the choice of settings depends on the precise situation and needs careful a priori tuning. The relationship between the sequence parameters, model parameters, and tissue MR properties is complex and needs to be understood in order for the scanner to be used most efficiently. This is of particular importance for powerful gradient systems such as MGH Connectom 10, which can span much larger space of sequence parameters, and can potentially probe small diameters in the brain that are not assessable with lower gradient strengths.

## Supporting information


**Figure S1.** Fixed b‐value comparison. Figure shows restricted signal *S*_*r*_(a) and its sensitivity to axon diameter $S_{r}^{\prime }$(a) for a ∈ {2; 10; 40} μm. The absolute values of *S*_r_ and $S_{r}^{\prime }$ are colour coded, with dark red being the highest value. The white lines are the lines with fixed b‐values b ∈ {300; 2500; 12500; 50000; 125000} s/mm^2^. Unit of $S_{r}^{\prime }$(a) is 1/μm.
**Figure S2.** A simple illustrative example of the impact n ⊥ G or fibre dispersion have on the diffusion signal. A range of sequences with *N* ∈ {1; 2; 3; 4; 5; 6} for a given gradient strength *G* = 300mT/m and pulse duration δ = 40ms are shown.
