# Supplementary material for: PGSE, OGSE, and sensitivity to axon diameter in diffusion MRI: Insight from a simulation study
Source: Magn Reson Med. 2015 Mar 25;75(2):688–700. doi: 10.1002/mrm.25631 (PMC4975609; doi:10.1002/mrm.25631)
Supplement: Supplementary file 1 — Figure S1. Fixed b‐value comparison. Figure shows restricted signal S r(a) and its sensitivity to axon diameter Sr′(a) for a ∈ {2; 10; 40} μm. The absolute values of S r and Sr′ are colour coded, with dark red being the highest value. The white lines are the lines with fixed b‐values b ∈ {300; 2500; 12500; 50000; 125000} s/mm2. Unit of Sr′(a) is 1/μm. Figure S2. A simple illustrative example of the impact n ⊥ G or fibre dispersion have on the diffusion signal. A range of sequences with N ∈ {1; 2; 3; 4; 5; 6} for a given gradient strength G = 300mT/m and pulse duration δ = 40ms are shown. [file MRM-75-688-s001.pdf]

1 Supporting Material

1.1 Fixed b-value

Here we relate some of the sensitivity results to the b-value, the widely used measure for quantifying the amount of diffusion weighting. The experiment simulates restricted signal $S_r(a)$ and its sensitivity $S'_r(a)$ for the range of sequence parameters in Λ introduced in the Results in the Main Text. Figure 1 shows the results for $a = 2\mu$m (top), $a = 10\mu$m (medium) and $a = 40\mu$m (bottom) for both $N = 1$ (left two columns) and $N = 2$ (right two columns). Note that an infinite slew rate is assumed for simplicity of the comparisons. White lines overlaid onto the figure are data for fixed b-values $b \in \{300, 2500, 12500, 50000, 125000\}$ s/mm^2.

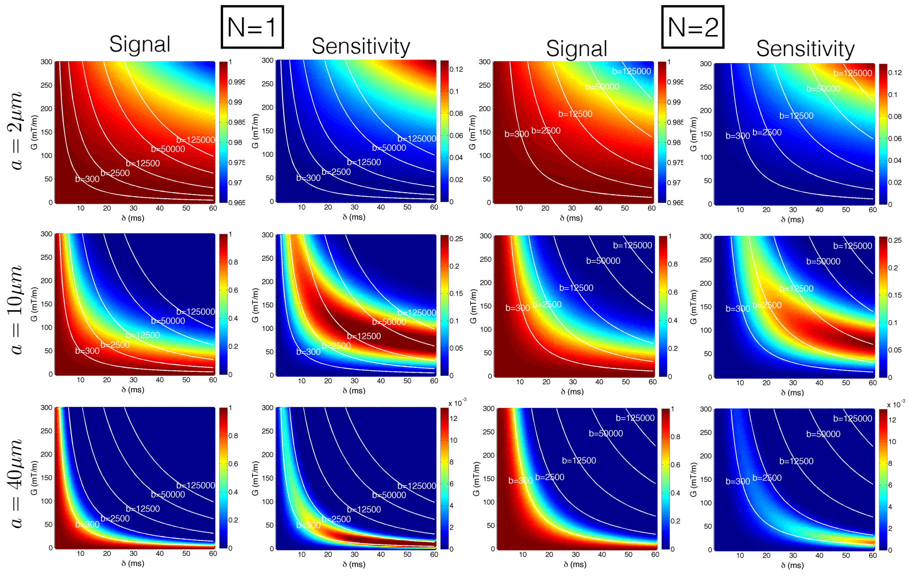

Figure 1: Fixed b-value comparison. Figure shows restricted signal $S_r(a)$ and its sensitivity to axon diameter $S'_r(a)$ for $a \in \{2, 10, 40\}\mu$m. The absolute values of S_r and S'_r are colour coded, with dark red being the highest value. The white lines are the lines with fixed b-values $b \in \{300, 2500, 12500, 50000, 125000\}$ s/mm^2. Unit of $S'_r(a)$ is $1/\mu$m.

The figure shows that, in the case of restriction, different combinations of δ, G and N can produce the same b-value while achieving very different signal attenuation and sensitivity to axon diameter. This can be seen from how aligned the plot isolines are with respect to the fixed b-value lines (b-lines). For example, for axon diameters of $40\mu m$, which provide almost free diffusion environment for the set of parameters considered here, isolines and b-lines are

almost perfectly aligned. On the other hand, for smaller diameters, the b-lines and the isolines are misaligned, suggesting that in the presence of strong restriction, signal attenuation depends on complex combinations of δ, G and N that can be very different from the particular combination defined by the b-value. The results for both $N = 1$, $N = 2$ and larger N (data not shown) all support the same conclusions. At present, when reporting the settings of a diffusion MRI experiment, b-value is commonly given without providing the detailed settings for the diffusion gradients. This figure shows that this practice is inadequate in the presence of significant restrictions.

1.2 Fixed G and δ

Here we show a simple illustrative example of the impact $\theta > 0$ and fibre dispersion have on the diffusion signal for $N = 1$ and $N > 1$ cases when both gradient strength and the pulse duration are fixed. Figure 2 shows a range of sequences with $N \in \{1, 2, 3, 4, 5, 6\}$ for a given gradient strength $G = 300\text{mT/m}$ and pulse duration $\delta = 40\text{ms}$. Note that an infinite slew rate is assumed for simplicity of the comparisons. When the $\theta = 0$ the sensitivity to axon diameter of different $N's$ is very similar. However, when $\theta > 0$ (the middle row) or fibres are dispersed (the right row) the sequence with the lowest b-value, $N = 6$, is the most sensitive, i.e. has the largest slope.

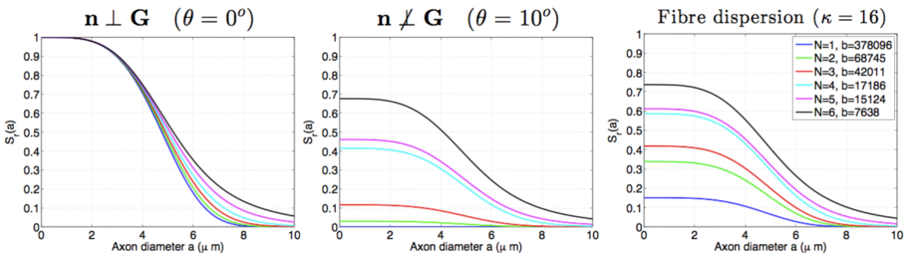

Figure 2: A simple illustrative example of the impact $\mathbf{n} \perp \mathbf{G}$ or fibre dispersion have on the diffusion signal. A range of sequences with $N \in \{1, 2, 3, 4, 5, 6\}$ for a given gradient strength $G = 300\text{mT/m}$ and pulse duration $\delta = 40\text{ms}$ are shown.
